# Transplantation outcomes in Philadelphia-positive acute lymphoblastic leukemia with additional cytogenetic abnormalities: A study on behalf of the Acute Leukemia Working Party of the European Society for Blood and Marrow Transplantation

**DOI:** 10.1038/s41409-026-02970-6

**Published:** 2026-07-13

**Authors:** Arnon Nagler, Jordi Esteve, Jacques-Emmanuel Galimard, Depei Wu, Erlie Jiang, Nathalie Dhedin, Anne Sirvent, Jan Vydra, Jakob Passweg, Bruno Lioure, Jaime Sanz, Jurjen Versluis, Yajing Xu, Cristina Castilla-Llorente, Rodrigo Martino Bufarull, Victoria Potter, Nour Ben Abdeljelil, Eolia Brissot, Ali Bazarbachi, Bipin Savani, Sebastian Giebel, Mohamad Mohty, Fabio Ciceri

**Affiliations:** 1 Division of Hematology, Sheba Medical Center, Tel Hashomer, and Tel Aviv University, Tel Aviv, Israel; 2 Hematology Department, Institute of Cancer & Blood Disorders (ICAMS), Hospital Clinic, IDIBAPS, University of Barcelona, Barcelona, Spain; 3 EBMT Paris Study Unit, Paris, France; 4 First-Affiliated Hospital of Soochow University, Suzhou, China; 5 Institute of Hematology, Chinese Academy of Medical Sciences, Tianjin, China; 6 Hematology Unit for Adolescents and Adults, Saint Louis Hospital, Paris, France; 7 CHU Lapeyronie, Montpellier, France; 8 Institute of Hematology and Blood Transfusion, Prague, Czechia; 9 University Hospital Basel, Basel, Switzerland; 10 ICANS - Institut de cancerologie Strasbourg Europe, Strasbourg, France; 11 Hematology Department, Hospital Universitari i Politècnic La Fe, Valencia Departament de Medicina Universitat de Valencia, CIBERONC, Instituto Carlos III, Madrid, Spain; 12 Erasmus MC Cancer Institute, Rotterdam, The Netherlands; 13 Central South University - Xiangya Hospital, Changsha, China; 14 Gustave Roussy Cancer Campus, Villejuif, France; 15 Hospital Santa Creu i Sant Pau, Barcelona, Spain; 16 Kings College Hospital, London, United Kingdom; 17 Centre National de Greffe de Moelle, Tunis, Tunisia; 18 Service d’Hématologie clinique et thérapie cellulaire, Hôpital Saint-Antoine, APHP, Paris, France; 19 Hematology-Oncology Division, Department of Internal Medicine, American University of Beirut Medical Center, Beirut, Lebanon; 20 Vanderbilt University Medical Center, Nashville, TN USA; 21 Maria Sklodowska-Curie National Research Institute of Oncology, Gliwice Branch, Gliwice, Poland; 22 Sorbonne University, Department of Haematology, Saint Antoine Hospital; INSERM UMR 938, Paris, France; 23 Ospedale San Raffaele, Haematology and BMT, Milano, Italy

**Keywords:** Leukaemia, Cancer genetics

## Abstract

We compared the presence of additional chromosomal abnormalities (ACAs, *n* = 345) with a referent group with no ACAs (*n *= 169) (noACAs) in patients with Ph+ ALL receiving TKI before transplantation. The groups did not differ significantly in age, gender, or interval from diagnosis to transplantation. More patients with ACAs received PB grafts (86.4% vs. 78.1%, *p* = 0.03), while more patients in the noACAs group received myeloablative conditioning (87% vs. 75.9%, *p *= 0.003). Day 30 ANC (≥10^9^/L) was 97.9% vs. 98.2%, and day 60 platelet count (≥20 × 10^9^/L) was 93.9% vs. 95.5%. Day 100 acute GVHD grades II-IV was 26.4% vs. 31%, and of grades III-IV was 8.6% vs. 9.7%. 4-year chronic GVHD was 40.5% vs. 39.9%, and the proportion of extensive chronic GVHD was 19.3% vs. 20.1%. 4-year NRM was 13.1% vs. 13.5%, and 4-year RI was 19.2% vs. 21.1%; 4-year LFS and OS were 67.7% vs. 65.4% and 76.3% vs. 78.5%. 4-year GRFS was 48.5% vs. 45%, respectively. In multivariable analysis, having ACAs and the number of ACAs did not significantly affect transplant outcomes. Administration of a TKI upfront, followed by transplantation, may overcome the poor prognostic influence of ACAs in Ph+ ALL.

## Introduction

Philadelphia chromosome-positive (Ph+) acute lymphoblastic leukemia (ALL) is a distinct subtype of ALL that has historically been associated with a very poor prognosis [1]. Allogeneic hematopoietic stem cell transplantation (HSCT) has improved the 5-year overall survival (OS) rate to ~44% and has been considered the only potentially curative treatment option for these patients [2]. Tyrosine kinase inhibitors (TKIs), initially imatinib and then second and third-generation TKIs nilotinib, dasatinib, and ponatinib, revolutionized the Ph+ ALL treatment paradigm, significantly improving patient outcomes [3–5]. The introduction of immunotherapies, including blinatumomab, inotuzumab ozogamicin, and chimeric antigen receptor T-cell (CAR-T) therapy, further improved outcomes, questioning the role of HSCT in Ph+ ALL [1, 6–9]. Nevertheless, HSCT is still fully indicated in patients with high-risk Ph+ ALL not achieving upfront measurable residual disease (MRD) negativity or those with unfavorable genetics like IKZF mutation or CDKN2A/2B gene deletion [6, 10–13]. Additional adverse cytogenetic abnormalities (ACAs) are detected in ~60–70% of patients with Ph+ ALL [14–18]. The presence of abnormalities such as −7, +8, 9/9p, hypodiploidy (<44 chromosomes), variant Ph translocations involving three or more chromosomes, or a complex karyotype (CK) defined by ≥5 chromosomal abnormalities has been associated with poor prognosis, although some variability exists among studies [14–16]. The impact of ACAs on prognosis in Ph+ ALL patients treated with a TKI is somewhat more controversial. The Japanese Adult Leukemia Study Group analyzed 206 Ph+ ALL patients being treated with a TKI, 63.6% of them harbored ACAs. In the multivariable analysis (MVA), coexistence of +der (22)t(9;22) and a CK was a significant risk factor for both OS and leukemia-free survival (LFS) [17]. Similarly, the MD Anderson group analyzed 152 Ph+ ALL treated with chemotherapy plus a TKI, comparing the outcome of 118 Ph+ ALL patients with ACAs to outcomes of 34 Ph+ ALL patients without ACAs [18]. Patients with one or more poor-risk ACAs (+der (22)t(9;22) and/or −9/9p) in the absence of high hyperdiploidy had significantly shorter relapse-free survival (RFS) and OS. Of note, poor-risk ACAs were prognostic in patients who received imatinib and dasatinib but not in those who received ponatinib [18]. The number of ACAs and clones and the presence of CK may also impact prognosis, as was demonstrated by Jain et al., analyzing 114 Ph+ ALL patients, demonstrating that patients with variant-/complex- t (9;22) had poorer OS and RFS and that patients with ≥2 clones had worse OS and RFS than patients with 1 clone [19]. The impact of ACAs on HSCT outcome in Ph+ ALL patients and especially in patients receiving TKIs pre- and or post- HSCT is even more controversial. Aldoss et al. reported a small cohort of 78 Ph+ ALL patients, with available data on conventional cytogenetics at diagnosis, treated with a TKI and HSCT. ACAs were observed in 53% of the patients. Three-year LFS and OS were inferior in the Ph+ ALL patients with ACAs compared to the Ph+-only cohort [20]. In contrast, Akahoshi et al. retrospectively evaluated 1375 Ph+ ALL patients who underwent their first HSCT in the TKI era, comparing transplantation outcomes of the 224 patients who had ACAs (16.3%) to those without ACAs [21]. The authors observed no difference in OS, relapse incidence (RI), or the risk of overall mortality at 4 years between patients with or without ACAs [21]. More recently, Zheng et al. retrospectively analyzed 136 patients with Ph+ ALL, evaluating the effect of ACAs on HSCT outcomes [22]. ACAs were observed in 44% of the patients, 5% of them were defined as major-route and included: +der (22), +der (9), + 8, −7, and CK. In the MVA, 3-year RI and PFS were significantly worse in Ph + ALL patients with + der (22), + 8, −7, and CK compared with patients with no ACAs. In addition, 3-year OS was significantly lower in Ph + ALL patients with −7 [22]. Given this uncertain impact of ACAs on transplant outcome in Ph+ ALL in the TKI era, we performed a contemporary registry-based, real-life study using data of the Acute Leukemia Working Party of the European Society for Blood and Marrow Transplantation (EBMT), comparing the outcomes of HSCT in Ph+ ALL patients receiving a TKI with ACAs to those of comparable patients with no ACAs.

## Patients and methods

### Study design and data collection

This was a retrospective, multicenter analysis using the dataset of the Acute Leukemia Working Party of the EBMT. The EBMT is a voluntary working group of more than 600 transplant centers that are required to report all consecutive stem cell transplantations and follow-ups once a year. EBMT minimum essential data forms are submitted to the registry by transplant center personnel following written informed consent from patients per the centers’ ethical research guidelines. All methods were performed in accordance with the relevant guidelines and regulations. Accuracy of data is assured by individual transplant centers and by quality control measures such as regular internal and external audits. The results of disease assessments at HSCT were also submitted and form the basis of this report. The study was conducted in accordance with the STROBE recommendations. Eligibility criteria for this analysis included adult patients ≥18 years of age with B Ph+ ALL in CR1 with full karyotyping available at the time of diagnosis, receiving a TKI pre-HSCT, and who underwent a first HSCT without previous autologous HSCT between 2010 and 2022. All donor types and cell sources were included. Data collected included recipient and donor characteristics (age, gender, and cytomegalovirus serostatus), recipient, disease characteristics, full karyotyping including t(9;22) involving one or more partners, addition derivative t(9;22), ACAs, number (addition derivative t(9;22) was not counted in the number of additional abnormalities) and type of abnormalities, number and type of monosomies and trisomies, IKZF1 or CDKN2A/B, P53 and others, type pf pre-HSCT TKI, pre-HSCT MRD status, year of transplant, type of conditioning regimen, stem cell source, and graft-versus [vs.]-host disease (GVHD) prophylaxis regimen. MRD status was assessed before transplantation at the individual centers using molecular techniques and/or immunophenotyping and was classified as either positive or negative. PCR was used in 79% of cases, flow-cytometry in 17% and a combination of PCR and flow-cytometry in 4% of cases. The conditioning regimen was defined as myeloablative (MAC) or reduced intensity (RIC) based on the reports from individual transplant centers as per previously established criteria [23]. The conditioning regimen was defined as MAC when containing total body irradiation (TBI) with a dose >6 Gray or a total dose of busulfan (Bu) > 8 mg/kg or >6.4 mg/kg when administered orally or intravenously, respectively, or a total dose of treosulfan ≥36 gr/m^2^. All other regimens were defined as RIC [23]. Regimens for GVHD prophylaxis were per institutional protocols. Grading of acute (a) and chronic (c) GVHD was performed using established criteria [24, 25]. The list of institutions contributing data to this study is provided in the Supplementary Appendix.

### Statistical analysis

Median values and interquartile ranges (IQR) were used for quantitative variables, and frequencies and percentages were used for categorical variables. The study endpoints were OS, LFS, RI, non-relapse mortality (NRM), and GVHD-free, relapse-free survival (GRFS), all assessed at 4 years, neutrophil and platelet recovery, aGVHD assessed at day 100, and cGVHD assessed at 4 years. All endpoints were measured from the time of transplantation. Day 30 neutrophil recovery was defined as achieving an absolute neutrophil count of ≥0.5 × 10^9^/L for three consecutive days. Day 60 platelet recovery was defined as achieving a platelet count of ≥20 × 10^9^/L for three consecutive days. TKI maintenance was defined as TKI given after HSCT in the absence of hematological relapse. OS was defined as the time to death from any cause. LFS was calculated from the day of HSCT until disease recurrence, death from any cause, or last follow-up. NRM was defined as death from any cause without previous relapse or progression. Patient-, disease-, and transplant-related characteristics were compared between the groups according to the presence or absence of the ACAs, using the Mann–Whitney U test for quantitative variables, and the chi-squared or Fisher’s exact test for categorical variables. The probabilities of OS, LFS, and GRFS were calculated using the Kaplan–Meier estimator [26]. Neutrophil and platelet recovery, aGVHD, cGVHD, RI, and NRM were calculated using cumulative incidences in a competing risk setting, with death in the absence of relapse being treated as a competing event for relapse. Death was considered a competing event for engraftment and TKI maintenance. To estimate the cumulative incidence of aGVHD or cGVHD, relapse, and death were considered as competing events. The median follow-up was estimated using reverse Kaplan-Meier estimation.

The impact of ACAs, i.e., 1 ACA, 2–3 ACAs, and ≥4 ACAs vs. absence of ACAs on outcomes was estimated using the Cox proportional-hazards regression model [26], adjusting for MRD, donor type, use of TBI associated with regimen intensity, female donor to male recipient, age, year of HSCT, and time from diagnosis to HCT. Results are expressed as the hazard ratio (HR) with a 95% confidence interval (95% CI).

All *p*-values were two-sided with a type 1 error rate fixed at 0.05. Statistical analyses were performed with R 4.2.3 (R Core Team, 2020). R: A language and environment for statistical computing. R Foundation for Statistical Computing, Vienna, Austria. URL 8832 https://www.R-project.org/ [27, 28].

## Results

### Patient, disease, and transplant characteristics

Five hundred and fourteen Ph+ ALL patients met the study inclusion criteria, 345 with ACAs (ACAs) and 169 with no ACAs (noACAs) (Table 1). Of the 345 patients with ACAs, 109 had 1 ACA, 100 had 2-3 ACAs, and 136 had ≥4 ACAs. An additional derivative was observed in 36.5% vs 4.7% of the ACAs and noACAs patients, respectively. One hundred and thirty-five patients (39.1%) had at least one monosomy, most frequently monosomy 7 (18.3%, *n *= 63), and 153 (44.3%) patients had at least one trisomy. Fourteen patients had chromosome 11 abnormalities, including two with 11q23 translocations. IKZF1 mutations were observed in 8.7% and 7.7% of patients with and without ACAs, while CDKN2A/B was present in 0.6% vs. 1.8%, respectively (Table 1). Median follow-up was 4.2 years (95% CI, 3.9–4.7) (Table 2). The median year of transplantation overall was 2018 (IQR, 2015–2020), and was 2018 in each group. Median age was 43.2 (IQR, 32.1–53.7) vs. 41 (IQR, 30.4–52.1) years (*p *= 0.14), and 55.7% vs. 52.1% were male in the ACAs vs. noACAs groups, respectively (*p *= 0.44). The most frequent TKI administered pre HSCT was imatinib in 45.5% vs. 36.7%, followed by dasatinib in 15.9% vs. 26.6%, or nilotinib in 11.3% vs. 10.1%, respectively (Supplementary Table S1). The presence of ACAs was associated with a significantly higher rate of MRD-negativity pre HSCT (56.2% vs. 46.5%, respectively, *p *= 0.04) (Table 2). Median time from diagnosis to HSCT was 5.8 months in both groups. Donors were matched siblings in 32.5% vs. 28.4%, unrelated in 37.1% vs. 27.2%, or haploidentical in 28.7% vs. 40.2%, in the ACAs and noACAs groups, respectively (Table 2). Patient CMV seropositivity was less frequent in the ACAs than in the noACAs group (70.5% vs. 57.2%, *p *= 0.009). Donor gender, patient gender, and female donor to male recipient combination did not differ significantly between the groups. More patients with ACAs received peripheral blood grafts compared to the noACAs group (86.4% vs. 78.1%, *p *= 0.03). The use of MAC was more frequent in noACAs than in ACAs patients (87% vs. 75.9%, *p *= 0.003) (Table 2). The most frequent conditioning in both groups was TBI-based (51.9% vs. 40.2%). Busulfan/cyclophosphamide was the most frequent chemotherapy-based regimen, 20% vs. 34.3%, respectively (Supplementary Table S2). Regarding GVHD prophylaxis, cyclosporine A (CSA) /methotrexate was used in 32.1% vs. 33.7%, and CSA/mycophenolate mofetil in 17.6% vs. 11.4%, respectively (Supplementary Table S3). Post-transplant cyclophosphamide was administered to 12.9% vs. 14.8%, respectively (*p *= 0.55), and use of anti-thymocyte globulin (ATG) was similar between the groups at 58.6% vs. 66.7%, respectively (*p *= 0.09) (Table 2).

**Table 1:** Additional chromosomal aberrations.

Variables	Modalities	*N *= 514	noACAs	ACAs
			*n *= 169	*n *= 345
t (9;22) involving one or more partners	t (1;9;22)	2	0	2
	t (3;9;22)	2	1	1
	t (6;13;9;22)	1	1	0
	t (6;17;9;22)	1	0	1
	t (6;9;22)	1	1	0
	t (7;9;22)	2	2	0
	t (9;22;10)	2	1	1
	t (9;22;11)	2	1	1
	t (9;22;14)	1	0	1
	t (9;22;16)	1	0	1
	t (9;22;21)	1	0	1
Addition derivative t (9;22)	No	380 (73.9)	161 (95.3)	219 (63.5)
	Yes	134 (26.1)	8 (4.7)	126 (36.5)
Number of additional abnormalities	No additional abn	169 (32.9)	169 (100)	
	1 add abns	109 (21.2)		109 (31.6)
	2–3 add abns	100 (19.5)		100 (29)
	≥4 add abns	136 (26.5)		136 (39.4)
Number of monosomies	0			210 (60.9)
	1–4			131 (38)
	5 or more			4 (1.2)
Number of trisomies (and additional Y)	0			192 (55.7)
	1–4			101 (29.3)
	5 or more			52 (15.1)
IKZF1 or CDKN2A/B	No	471 (91.6)	156 (92.3)	315 (91.3)
	Yes	43 (8.4)	13 (7.7)	30 (8.7)
IKZF1	No	471 (91.6)	156 (92.3)	315 (91.3)
	Yes	43 (8.4)	13 (7.7)	30 (8.7)
CDKN2A/B	No	509 (99)	166 (98.2)	343 (99.4)
	Yes	5 (1)	3 (1.8)	2 (0.6)
TP53	No	514 (100)	169 (100)	345 (100)
Monosomy 7	No			282 (81.7)
	Yes			63^a^ (18.3)
ABN7 (not t, tri or mono)	No			322 (93.3)
	Yes			23 (6.7)
DEL7Q	No			341 (98.8)
	Yes			4^a^ (1.2)
MONO7 or DEL7Q	No			279 (80.9)
	Yes			66^a^ (19.1)
Monosomy 9	No			318 (92.2)
	Yes			27 (7.8)
DEL9Q	No			340 (98.6)
	Yes			5 (1.4)
MONO or DEL9Q	No			313 (90.7)
	Yes			32 (9.3)
ABN11Q23 (including the translocations)	No			343 (99.4)
	Yes			2 (0.6)
Monosomy X (or X full deletion of male)	No			339 (98.3)
	Yes			6 (1.7)
Full deletion of Y	No			337 (97.7)
	Yes			8 (2.3)

*ACAs* additional chromosomal abnormalities, *noACAs* no additional chromosomal abnormalities, *abn(s)* abnormality/abnormalities, *add* additional, *tri* trisomy, *mono* monosomy.

Results expressed as frequency (%).

^a^One patient harbored both monosomy 7 and del(7q).

**Table 2:** Patient and transplant characteristics.

Variables	Modalities	Total population *N *= 514	noACAs	ACAs	*P*-value
			*n *= 169	*n *= 345	
Median FU (y)		4.2 (3.9–4.7)	4.2 (3.7–4.9)	4.3 (3.7–4.9)	
Years of HSCT	Median [IQR]	2018 [2015–2020]	2018 [2016–2021]	2018 [2015–2020]	0.27
	(Range)	(2010–2022)	(2010–2022)	(2010–2022)	
Age at HSCT	Median [IQR]	42.6 [31.7–53.5]	41 [30.4–52.1]	43.2 [32.1–53.7]	0.14
	(Range)	(18.1–76.1)	(18.1–70.1)	(18.3–76.1)	
Months between diagnosis and HSCT	Median [IQR]	5.8 [4.7–7.1]	5.8 [4.8–7]	5.8 [4.7–7.2]	0.63
	(Range)	(0.4–29.6)	(2.3–29.6)	(0.4–20.5)	
	Missing	3	0	3	
Patient gender	Female	234 (45.5)	81 (47.9)	153 (44.3)	0.44
	Male	280 (54.5)	88 (52.1)	192 (55.7)	
Donor gender	Female	176 (34.2)	60 (35.5)	116 (33.6)	0.67
	Male	338 (65.8)	109 (64.5)	229 (66.4)	
Female donor to male recipient	No	434 (84.4)	148 (87.6)	286 (82.9)	0.17
	Yes	80 (15.6)	21 (12.4)	59 (17.1)	
Source of cells	PB	430 (83.7)	132 (78.1)	298 (86.4)	0.03
	BM	38 (7.4)	14 (8.3)	24 (7)	
	BM + PB	46 (8.9)	23 (13.6)	23 (6.7)	
Donor type	MSD	160 (31.1)	48 (28.4)	112 (32.5)	ND
	MOR	3 (0.6)	2 (1.2)	1 (0.3)	
	Syngeneic	1 (0.2)	0 (0)	1 (0.3)	
	MMR 1 locus	4 (0.8)	1 (0.6)	3 (0.9)	
	Haplo	167 (32.5)	68 (40.2)	99 (28.7)	
	MMR (missing HLA)	5 (1)	4 (2.4)	1 (0.3)	
	UD 10/10	120 (23.3)	34 (20.1)	86 (24.9)	
	UD 9/10	32 (6.2)	7 (4.1)	25 (7.2)	
	UD < = 8/10	5 (1)	1 (0.6)	4 (1.2)	
	UD	17 (3.3)	4 (2.4)	13 (3.8)	
Patient CMV	Negative	168 (38.8)	38 (29.5)	130 (42.8)	0.009
	Positive	265 (61.2)	91 (70.5)	174 (57.2)	
	Missing	81	40	41	
Donor CMV	Negative	158 (39.6)	46 (41.4)	112 (38.9)	0.64
	Positive	241 (60.4)	65 (58.6)	176 (61.1)	
	Missing	115	58	57	
CMV donor: CMV patient	Neg: Neg	72 (18.1)	20 (18)	52 (18.2)	0.02
	Neg: Pos	85 (21.4)	26 (23.4)	59 (20.6)	
	Pos: Neg	85 (21.4)	13 (11.7)	72 (25.2)	
	Pos: Pos	155 (39)	52 (46.8)	103 (36)	
	Missing	117	58	59	
Myeloablative	No	105 (20.5)	22 (13)	83 (24.1)	0.003
	Yes	408 (79.5)	147 (87)	261 (75.9)	
	Missing	1	0	1	
PTCY	No	442 (86.5)	144 (85.2)	298 (87.1)	0.55
	Yes	69 (13.5)	25 (14.8)	44 (12.9)	
	Missing	3	0	3	
In vivo TCD	No	175 (34.9)	53 (31.5)	122 (36.6)	0.09
	ATG	307 (61.3)	112 (66.7)	195 (58.6)	
	Alemtuzumab	19 (3.8)	3 (1.8)	16 (4.8)	
	Missing	13	1	12	
MRD	Negative	263 (53.1)	73 (46.5)	190 (56.2)	0.04
	Positive	232 (46.9)	84 (53.5)	148 (43.8)	
	Missing	19	12	7	

*ACAs* additional chromosomal abnormalities, *noACAs* no additional chromosomal abnormalities, *FU* follow up, *IQR* interquartile range, *HSCT* hematopoietic stem cell transplantation, *y* years, *HLA* human leukocyte antigen, *MSD* matched sibling donor, *MOR* matched other related, *MMR* mismatched related, *Haplo* haploidentical, *UD* unrelated donor, *CMV* cytomegalovirus, *BM* bone marrow, *PB* peripheral blood, *TCD* T-cell depletion, *ATG* anti-thymocyte globulin, *PTCY* post transplantation cyclophosphamide, *MRD* measurable residual disease, *Neg* negative, *Pos* positive, *ND* not done.

Results expressed as frequency (%) unless otherwise stated.

### Transplantation outcomes

**Fig. 1:**
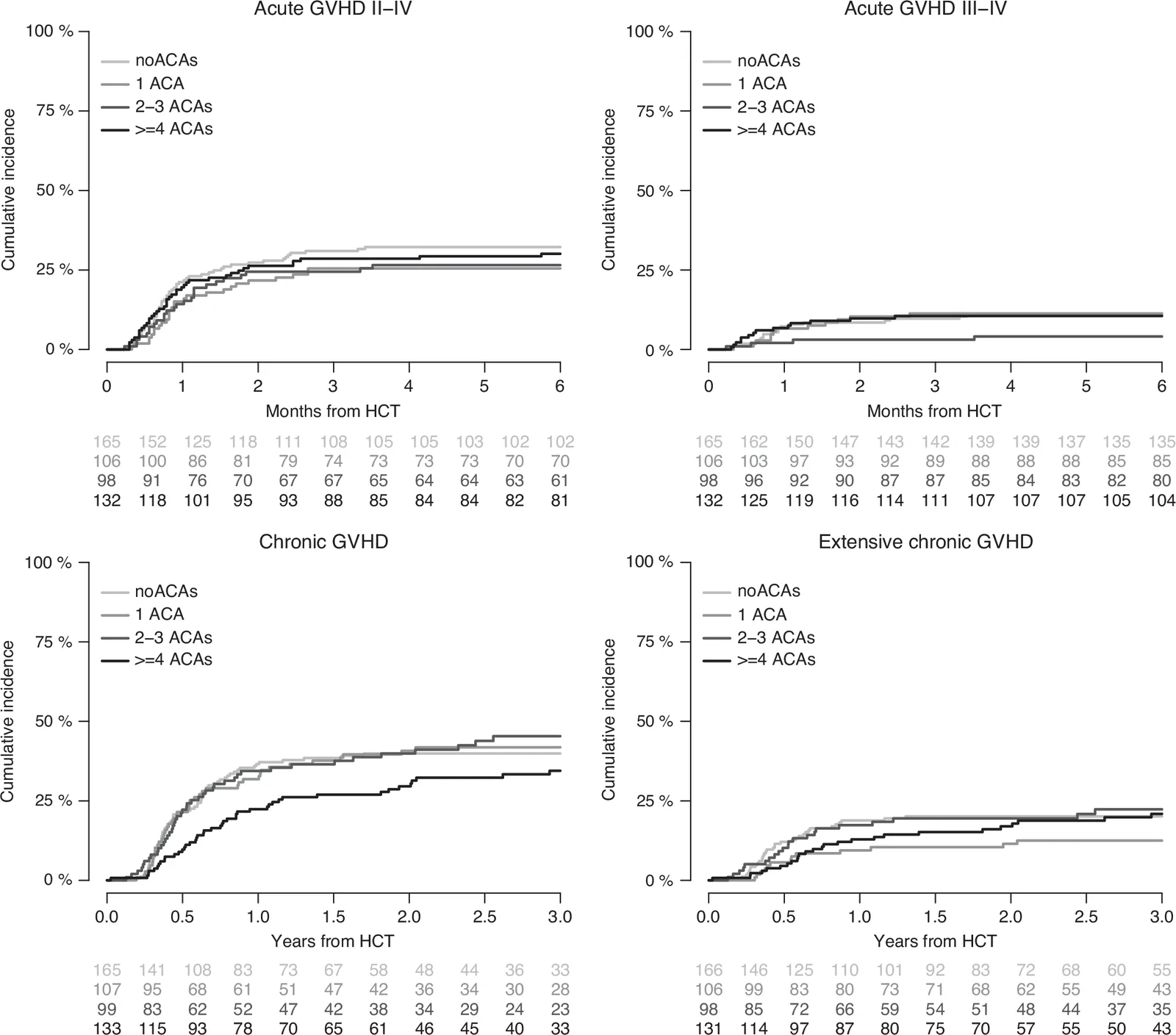
Hematopoietic stem cell transplant (HSCT) outcomes in Philadelphia positive (Ph+) acute lymphoblastic leukemia (ALL) patients with (NoACAs, 1 ACA, 2–3 ACAs, ≥4 ACAs) or NoACAs. Graft- versus-host disease (GVHD).

**Fig. 2:**
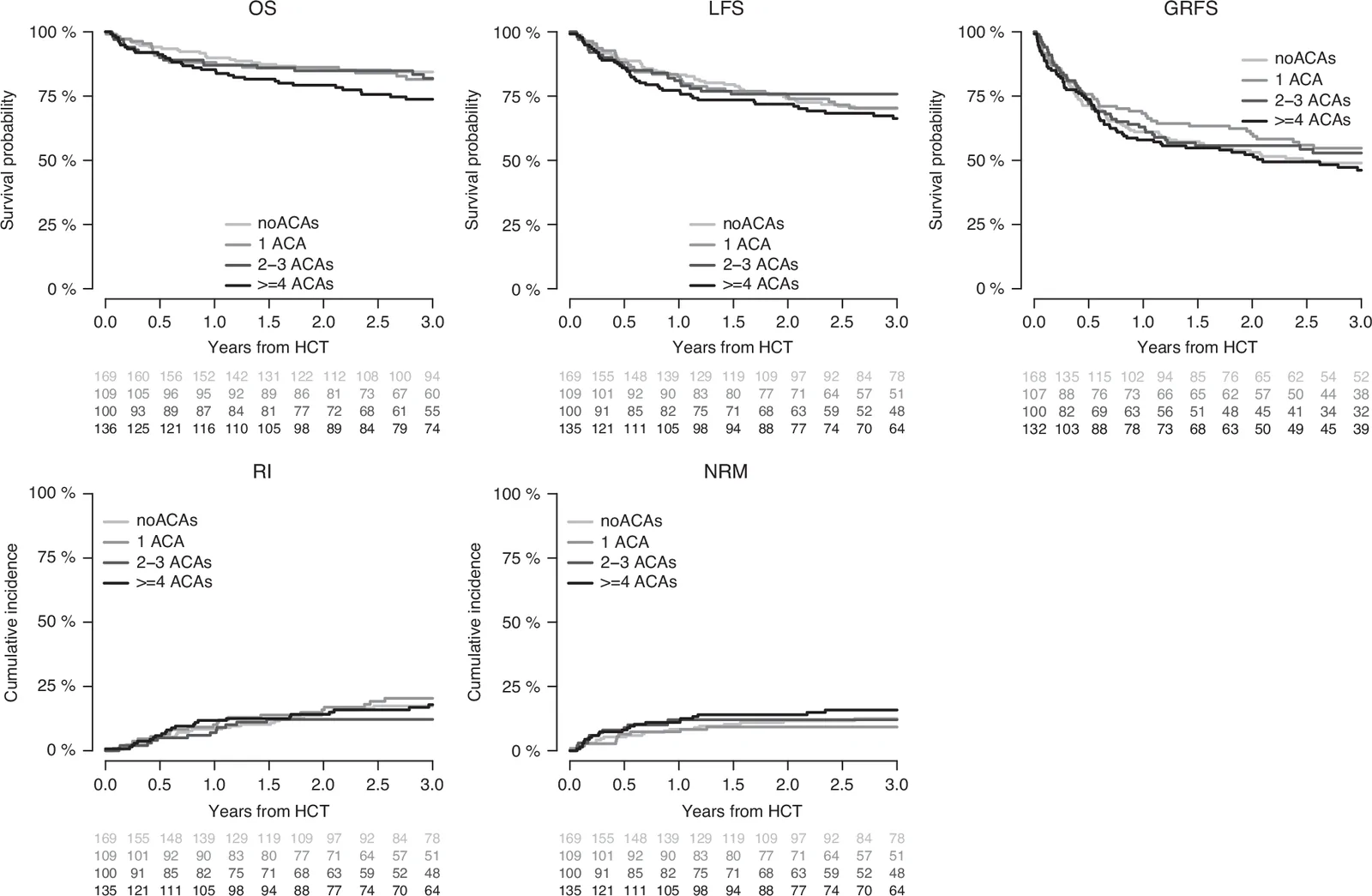
Hematopoietic stem cell transplant (HSCT) outcomes in Philadelphia positive (Ph+) acute lymphoblastic leukemia (ALL) patients with (1 ACA, 2–3 ACAs, ≥4 ACAs) or noACAs. Overall survival (OS), leukemia-free survival (LFS), graft-versus-host disease, relapse-free survival (GRFS), relapse incidence (RI), and non-relapse mortality (NRM).

Day 30 absolute neutrophil count recovery (≥0.5 × 10^9^/L) was 97.9% vs. 98.2%, and day 60 platelet recovery (>20 × 10^9^/L) was 93.9% vs. 95.5% in the ACAs and noACAs groups, respectively (Table 3). Cumulative incidence of TKI maintenance post HSCT was 29.5% and 38.4% overall; at 6 months it was 30.9% vs. 29.1%, and at 1 year it was 39.2% vs. 36.7%, for ACAs and noACAs, respectively (Supplementary Table S1B). Cumulative incidence of day 100 aGVHD Grade II-IV was 26.4% (95% CI 21.8–31.2%) vs. 31% (95% CI 24.1–38.1%), and for grade III-IV it was 8.6% (95% CI 5.9–11.9%) vs. 9.7% (95% CI 5.8–14.8%), in the ACAs and noACAs groups, respectively. Four-year, all grades cGVHD was 40.5% (95% CI 35–45.9%) vs. 39.9% (95% CI 32.3–47.3%), and 4-year extensive cGVHD was 19.3% (95% CI 15–23.9%) vs. 20.1% (95% CI 16.1–23.3%), respectively (Table 3; Fig. 1). Four-year NRM and RI were 13.1% (95% CI 9.7–17) vs. 13.5% (95% CI 8.6–19.4%), and 19.2% (95% CI 14.9–23.8%) vs. 21.1% (95% CI 14.8–28.3%), respectively (Table 3, Fig. 2). Four-year LFS, OS, and GRFS were 67.7% (95% CI 62.2–72.7%) vs. 65.4% (95% CI 56.9–72.6%), 76.3% (95% CI 71.2–80.7%) vs. 78.5% (95% CI 70.5–84.5%), and 48.5% (95% CI 42.7–54%) vs. 45.1% (95% CI 36.8–53%), in the ACAs vs. noACAs groups, respectively (Table 3, Fig. 1). In the MVA, none of the outcomes were significantly different in patients with ACAs 1, 2–3, and ≥4 abnormalities compared to patients with noACAs, respectively (Table 4; Supplementary Figs. 1, 2). Additionally, monosomy 7/del[7q] and derivative t (9;22) did not affect transplantation outcomes in univariate analyses (Supplementary Table S4). Abnormality 9 and trisomy 8 were also evaluated in the univariate analysis, and neither was associated with significant differences in OS, LFS, RI, or NRM (data not shown).

**Table 3:** Transplantation outcomes.

		All patients	noACAs	ACAs
Outcomes	*N*	Estimation (95% CI)	Estimation (95% CI)	Estimation (95% CI)
OS (4 y)	514	77 (72.7–80.6)	78.5 (70.5–84.5)	76.3 (71.2–80.7)
LFS (4 y)	514	67 (62.4–71.2)	65.4 (56.9–72.6)	67.7 (62.2–72.7)
RI (4 y)	514	19.8 (16.2–23.7)	21.1 (14.8–28.3)	19.2 (14.9–23.8)
NRM (4 y)	514	13.2 (10.4–16.4)	13.5 (8.6–19.4)	13.1 (9.7–17)
Poly recovery (30 d)	510	98 (96.3–99)	98.2 (93.9–99.5)	97.9 (95.6–99)
Platelet recovery (>=20) (60 d)	482	94.5 (91.9–96.2)	95.5 (90.5–97.9)	93.9 (90.5–96.2)
aGVHD-II/IV (100 d)	502	27.9 (24.1–31.9)	31 (24.1–38.1)	26.4 (21.8–31.2)
aGVHD-III/IV (100 d)	502	9 (6.7–11.7)	9.7 (5.8–14.8)	8.6 (5.9–11.9)
cGVHD (4 y)	505	40.4 (36–44.8)	39.9 (32.3–47.3)	40.5 (35–45.9)
cGVHD Ext (4 y)	502	19.6 (16.1–23.3)	20.1 (14.4–26.6)	19.3 (15–23.9)
GRFS (4 y)	508	47.3 (42.6–51.9)	45.1 (36.8–53)	48.5 (42.7–54)

*ACAs* additional chromosomal abnormalities, *noACAs* no additional chromosomal abnormalities, *y* years, *d* days, *RI* relapse incidence, *NRM* non-relapse mortality, *LFS* leukemia-free survival, *OS* overall survival, *aGVHD* acute graft-versus-host disease, *cGVHD* chronic graft-versus-host disease, *Ext* extensive, *Poly* polymorphonuclear, *CI* confidence interval, *GRFS* GVHD free, relapse free survival, *CI* confidence interval.

**Table 4:** Multivariable analysis.

Variables	Modalities	LFS		OS		RI		NRM		GRFS	
		HR (95% CI)	*p* value	HR (95% CI)	*p* value	HR (95% CI)	*p* value	HR (95% CI)	*p* value	HR (95% CI)	*p* value
Additional abnormalities	noACAs	1		1		1		1		1	
	1 ACA	0.93 (0.60–1.46)	0.76	1.09 (0.63–1.88)	0.77	1.12 (0.64–1.95)	0.69	0.70 (0.33–1.52)	0.37	0.87 (0.6–1.25)	0.44
	2–3 ACAs	0.90 (0.57–1.43)	0.65	0.95 (0.53–1.71)	0.86	0.80 (0.43–1.49)	0.49	1.09 (0.54–2.21)	0.81	0.95 (0.66–1.36)	0.77
	≥4 ACAs	1.06 (0.70–1.59)	0.78	1.36 (0.83–2.24)	0.22	0.87 (0.51–1.51)	0.63	1.39 (0.75–2.57)	0.3	0.97 (0.69–1.36)	0.85
MRD	Negative	1		1		1		1		1	
	Positive	1.39 (1.01–1.91)	**0.04**	1.54 (1.04–2.27)	**0.03**	1.20 (0.79–1.82)	0.39	1.68 (1.02–2.76)	**0.04**	1.2 (0.92–1.55)	0.17
Donor type	Matched rel donor	1		1		1		1		1	
	Mismatched rel donor	0.84 (0.51–1.39)	0.5	1.24 (0.67–2.29)	0.49	0.41 (0.20–0.83)	**0.01**	2.24 (1.02–4.92)	**0.04**	0.64 (0.43–0.98)	0.04
	UD	1.22 (0.85–1.75)	0.29	1.34 (0.85–2.1)	0.2	0.97 (0.62–1.53)	0.9	1.91 (1.04–3.52)	**0.04**	1.01 (0.75–1.36)	0.92
Conditioning regimen	MAC-TBI	1		1		1		1		1	
	MAC-chemo	1.10 (0.70–1.73)	0.68	1.19 (0.69–2.07)	0.53	1.27 (0.70–2.29)	0.44	1.03 (0.52–2.06)	0.93	1.08 (0.75–1.56)	0.67
	Non-MAC	1.02 (0.66–1.56)	0.94	0.91 (0.54–1.52)	0.72	1.49 (0.85–2.62)	0.16	0.60 (0.30–1.19)	0.14	1.03 (0.72–1.49)	0.85
Female donor to male patient	No	1		1		1		1		1	
	Yes	0.99 (0.66–1.50)	0.96	0.93 (0.56–1.55)	0.78	1.12 (0.66–1.89)	0.67	0.84 (0.42–1.64)	0.6	1.14 (0.82–1.59)	0.43
Year at HSCT (HR for 5 y increment)		0.53 (0.41–0.69)	<**0.001**	0.41 (0.30–0.56)	<**0.001**	0.60 (0.43–0.85)	**0.004**	0.45 (0.30–0.68)	<**0.001**	0.63 (0.51–0.77)	<**0.001**
Age at HSCT (HR for 10 y increment)		1.17 (1.02–1.34)	**0.03**	1.29 (1.09–1.53)	**0.003**	1.07 (0.90–1.28)	0.45	1.32 (1.06–1.64)	**0.01**	1.05 (0.94–1.17)	0.43
Time from diag to HSCT	Less than 6 months	1		1		1		1		1	
	6 months or more	1.53 (1.10–2.12)	**0.01**	2.01 (1.34–3.01)	<**0.001**	1.21 (0.79–1.88)	0.38	2.00 (1.19–3.34)	**0.008**	1.36 (1.04–1.77)	**0.03**

Variables	Modalities	AGVH II-IV		AGVH III-IV		CGVH		CGVH ext	
		HR (95% CI)	*p* value	HR (95% CI)	*p* value	HR (95% CI)	*p* value	HR (95% CI)	*p* value
Additional abnormalities	noACAs	1		1		1		1	
	1 ACA	0.78 (0.47–1.28)	0.32	1.05 (0.49–2.23)	0.9	0.95 (0.63–1.44)	0.81	0.58 (0.29–1.16)	0.12
	2–3 ACAs	0.89 (0.53–1.47)	0.64	0.36 (0.12–1.09)	0.07	1.04 (0.68–1.57)	0.87	1.07 (0.58–1.96)	0.83
	≥4 ACAs	0.95 (0.60–1.51)	0.83	0.93 (0.43–1.98)	0.84	0.78 (0.51–1.19)	0.25	0.92 (0.5–1.69)	0.79
MRD	Negative	1		1		1		1	
	Positive	1.09 (0.77–1.55)	0.62	0.83 (0.46–1.52)	0.55	1.14 (0.84–1.53)	0.4	1.38 (0.88–2.16)	0.16
Donor type	Matched rel donor	1		1		1		1	
	Mismatched rel donor	1.40 (0.77–2.57)	0.27	1.58 (0.66–3.79)	0.31	0.81 (0.5–1.3)	0.37	0.37 (0.16–0.87)	0.02
	UD	1.58 (1.02–2.46)	**0.04**	1.41 (0.66–3.02)	0.38	0.76 (0.52–1.12)	0.17	0.8 (0.47–1.37)	0.42
Conditioning regimen	MAC-TBI	1		1		1		1	
	MAC-chemo	0.84 (0.49–1.44)	0.54	1.03 (0.47–2.28)	0.94	0.97 (0.63–1.51)	0.9	0.99 (0.51–1.94)	0.99
	Non-MAC	0.43 (0.24–0.78)	**0.005**	0.91 (0.38–2.21)	0.84	0.67 (0.42–1.09)	0.11	0.59 (0.29–1.2)	0.14
Female donor to male patient	No	1		1		1		1	
	Yes	0.86 (0.53–1.41)	0.56	0.78 (0.33–1.88)	0.59	1.53 (1.06–2.22)	**0.02**	1.63 (0.97–2.74)	0.07
Year at HSCT (HR for 5 y increment)		0.81 (0.61–1.08)	0.16	0.68 (0.42–1.09)	0.11	0.62 (0.49–0.80)	<**0.001**	0.52 (0.37–0.74)	<**0.001**
Age at HSCT (HR for 10 y increment)		0.91 (0.79–1.06)	0.22	0.96 (0.74–1.23)	0.73	1.03 (0.91–1.17)	0.63	1.05 (0.86–1.27)	0.65
Time from diag to HSCT	Less than 6 months	1		1		1		1	
	6 months or more	1.47 (1.01–2.14)	**0.04**	1.36 (0.74–2.48)	0.32	1.17 (0.84–1.62)	0.36	1.43 (0.88–2.33)	0.15

*ACAs* additional chromosomal abnormalities, *noACAs* no additional chromosomal abnormalities, *HSCT* hematopoietic stem cell transplantation, *y* year, *HR* hazard ratio, *IQR* interquartile range, *RI* relapse incidence, *NRM* non-relapse mortality, *LFS* leukemia-free survival, *OS* overall survival, *AGVH* acute graft-versus-host disease, *CGVH* chronic graft-versus-host disease, *ext* extensive, *GRFS* GVHD-free, relapse-free survival, *CI* confidence interval, *Poly* polymorphonuclear, *CMV* cytomegalovirus, *RIC* reduced intensity conditioning, *MAC* myeloablative conditioning, *rel* related, *UD* unrelated donor, *TBI* total body irradiation, *chemo* chemotherapy, *MRD* measurable residual disease, *diag* diagnosis. Bold values indicate statistical significance (*p* < 0.05).

### Impact of covariates

MRD-positivity pre-transplantation was associated with higher NRM with an HR  =  1.68 (95% CI 1.02–2.76, *p *= 0.04), worse LFS, HR  =  1.39 (95% CI 1.01–1.91, *p *= 0.04), and OS, HR  =  1.54 (95% CI 1.04–2.27, *p *= 0.03) (Table 4). Compared with matched related donors, mismatched related donors were associated with a lower risk of RI, HR  =  0.41 (95% CI 0.2–0.83, *p *= 0.01), while a matched unrelated donor was associated with a higher risk of grades II-IV aGVHD, HR  =  1.58 (95% CI 1.02–2.46, *p *= 0.04). Both mismatched and matched unrelated donors, compared to matched related donors, were associated with higher NRM, HR  =  2.24 (95% CI 10.2–4.92, *p *= 0.04), and HR  =  1.91 (95% CI 1.04–3.52, *p *= 0.04), respectively (Table 4). A non-MAC regimen was associated with a lower risk of grades II-IV aGVHD, HR  =  0.43 (95% CI 0.24–0.78, *p *= 0.005), while a female donor to male recipient combination was associated with a higher risk of total cGVHD, HR  =  1.53 (95% CI 1.06–2.22, *p *= 0.02). Year of HSCT (HR per 5-year increment) was associated with better LFS, OS, GRFS RI, NRM, total cGVHD, and extensive cGVHD (Table 4). Age at HSCT (HR per 10-year increment) was associated with worse LFS, OS, and NRM. Finally, a greater than 6-month interval from diagnosis to HSCT had a negative impact on LFS, OS, GRFS, NRM, and grades II-IV aGVHD (Table 4).

### Cause of death

A total of 120 patients (23.3%) died after transplant. Primary disease was the main cause, constituting 43.6% and 44% of the deaths in ACAs and noACAs, respectively. The second most common cause of death in the noACAs group was infection, 24% vs. 14.1% in the noACAs group, while in ACAs patients, it was GVHD, being 23.1% vs. 12% in the noACAs group (Table 4). Other transplant-related deaths accounted for 12.8% and 12%, respectively. Infrequent causes of death, such as secondary malignancies, veno-occlusive disease of the liver, interstitial pneumonitis, thrombotic microangiopathy, and central nervous system toxicity, were similar between the two groups (Table 5).

**Table 5:** Cause of death.

Cause of death	*N *= 120	noACAs	ACAs
		*n *= 36	*n *= 84
Relapse	45 (43.7)	11 (44)	34 (43.6)
GVHD	21 (20.4)	3 (12)	18 (23.1)
Infection	17 (16.5)	6 (24)	11 (14.1)
IP	2 (1.9)	1 (4)	1 (1.3)
Sec malignancy	1 (1)	0 (0)	1 (1.3)
TMA	1 (1)	0 (0)	1 (1.3)
VOD	2 (1.9)	1 (4)	1 (1.3)
CNS tox	1 (1)	0 (0)	1 (1.3)
HSCT-related other	13 (12.6)	3 (12)	10 (12.8)
missing	17	11	6

*ACAs* additional chromosomal abnormalities, *noACAs* no additional chromosomal abnormalities, *HSCT* hematopoietic stem cell transplantation, *GVHD* graft-versus-host disease, *VOD* veno-occlusive disease of the liver, *IP* interstitial pneumonitis, *Sec* secondary, *CNS* central nervous system, *tox* toxicity, *TMA* thrombotic microangiopathy.

Results expressed as frequency (%).

## Discussion

In the present study, which focused on Ph+ ALL patients with ACAs who received a TKI before transplantation, we evaluated the impact of ACAs on transplant outcomes compared with those of Ph+ ALL patients without ACAs in a real-world setting. Among a relatively large cohort of 514 patients, 345 (67%) harbored ACAs, a frequency consistent with that reported in previous studies [14–18]. We did not observe significant differences in any of the transplantation outcomes—including LFS, OS, GRFS, RI, NRM, myeloid and platelet engraftment, or the incidence of acute and cGVHD between patients with and without ACAs. Cytogenetic abnormalities are well-known independent prognostic factors for various hematologic disorders [29, 30], including Ph+ ALL [11, 15, 17, 18]; however, less is known about the impact of ACAs in such patients being treated with TKIs before an HSCT with TKI maintenance therapy. HSCT outcomes, including NRM, RI, LFS, OS, and GRFS, did not differ between Ph+ ALL patients with ACAs and those without ACAs. Furthermore, the number of ACAs made no impact. The lack of a deleterious prognostic value for ACAs in Ph+ ALL patients receiving TKIs and undergoing HSCT is important in view of most of the previous publications reporting a detrimental impact of ACAs at diagnosis on the prognosis of Ph+ ALL patients [11, 15, 17, 18]. Li et al. analyzed the significance of ACAs in 110 patients with Ph+ ALL receiving chemotherapy combined with imatinib. Patients with ACAs had a shorter disease-free survival and OS. Patients with loss of chromosomes 7, 7p, 9, and 9p had inferior outcomes compared to patients with other secondary aberrations and to those without [31]. Similarly, in a series of 80 adult patients with Ph+ ALL who were treated with chemotherapy including imatinib, Yanada et al. observed that the presence of ACAs was the only independent prognostic factor for RFS and was associated with a 2.8-fold increased risk of treatment failure (*p *= 0.027) [32]. However, it is conceivable that the prognostic impact of ACAs at diagnosis in Ph+ ALL differs between patients treated with chemotherapy with or without TKIs and those managed primarily with TKI-based therapy followed by HSCT, particularly when TKI maintenance therapy is administered post transplantation. This hypothesis formed the theoretical basis of our current study. As mentioned earlier, we observed no significant differences in any transplantation outcomes, including LFS, OS, GRFS, RI, NRM, myeloid and platelet engraftment, or the incidence of acute and cGVHD between patients with and without ACAs, including those with additional CKs. Previous results in this regard are indeed conflicting. In a relatively small cohort of patients treated with a TKI followed by HSCT, Aldoss et al. reported inferior 3-year LFS and OS among patients with Ph+ ALL and ACAs compared with those with Ph+ ALL without ACAs [20]. Similarly, x Zheng et al. found that patients with Ph+ ALL and major-route ACAs, including +8, −7, and complex karyotype (CK), as well as patients with +der [22], had a higher 3-year relapse incidence (RI) compared with Ph+ ALL patients without ACAs. Moreover, patients with +8, −7, and CK had inferior 3-year progression-free survival (PFS), while the presence of −7 was also associated with inferior 3-year overall survival (OS). The authors concluded that ACAs present at diagnosis have a significant prognostic impact on transplantation outcomes in patients with Ph+ ALL [22]. In contrast, and consistent with our results, a Japanese registry-based study retrospectively evaluated 1375 patients with Ph+ ALL (16.3% with ACAs) who underwent their first HSCT in the TKI era. In the MVA, no statistically significant differences were observed in the risk of overall mortality or risk of RI between the groups with and without ACAs. In addition, no difference was found in 4-year OS, and no specific ACA was associated with poor OS [21]. Finally, in a more recent report combining data from three Japanese Adult Leukemia Study Group prospective trials conducted in the TKI era, the authors analyzed 206 adults with newly diagnosed Ph+ ALL. Among these, 63.6% had ACAs, of which 48.1% exhibited a CK, defined as the presence of at least three abnormalities, i.e., t (9;22) plus two or more additional aberrations. HSCT in CR1 was performed in 67.0% of 206 patients. Although remission could be achieved at the same rate in Ph+ ALL patients with or without ACAs, the patients with ACAs were characterized by early relapse. In the MVA, while coexistence of +der (22)t(9;22) and a CK was a significant risk factor for both OS and LFS, +der (22)t(9;22) alone or a CK alone were not significant risk factors [17]. The somewhat discordant results obtained by all these studies may be due to diversity in patient populations with respect to age, individual and overall number of ACAs, treatment received, study period, and analysis of possible confounding factors such as interaction with co-mutations with a known prognostic value, including IKZF1 or CDKN2A/B, P53, and others [6–9].

Of note, we have demonstrated that the proportion of patients achieving MRD-negativity pre-HSCT (which in the MVA was associated with significantly lower NRM and superior LFS and OS) was significantly higher in patients with ACAs. This result is in line with the results of Aldoss et al. [20], who also reported that patients with ACAs had a lower rate of MRD-positivity pre-HSCT. This interesting finding may suggest that Ph+ ALL patients with ACAs achieve faster early molecular clearance; however, it may also reflect selection bias, as clinicians may regard patients with ACAs as high risk and therefore administer more intensive therapy. Moreover, both the number and the specific type of ACAs may influence prognosis differently. For example, loss-type abnormalities such as −7/7q− or −9/9p may confer a more adverse prognosis than gain-type lesions such as +8 or +der [18, 21, 22, 31, 33]. Short et al. evaluated the impact of individual ACAs, +der (22)t(9;22) and/or −9/9p in the absence of high hyperdiploidy, which was present in 16% of patients and constituted a poor-risk ACA group. Patients with ≥1 poor-risk ACA in the absence of hyperdiploidy had significantly shorter RFS and OS [18]. We did not find an association between the number of ACAs and post-transplantation outcomes. Interestingly, in the pre-TKI era, the Cancer and Leukemia Group B analyzed the impact of secondary cytogenetic changes in 111 newly diagnosed Ph+ ALL patients, demonstrating that ≥3 secondary aberrations were associated with a higher CR rate [16]. Motlló et al. reported a frequency of 24% for CK (≥5 abnormalities) with no differences in outcomes comparing patients with or without CKs [34]. Due to the small number of individual ACAs, we could not assess the association between any specific ACA and clinical outcomes, and could not compare the prognostic impact of one particular ACA with that of others. As mentioned above, the variability observed across studies is most likely attributable to heterogeneity in multiple factors. In addition, the wide time span covered by these studies represents an important confounding variable, as substantial improvements in outcomes have been achieved in more recent years owing to the introduction of novel therapeutic modalities and advances in supportive care [35].

The other prognostic factors we observed in the MVA, including year of HSCT, age, donor-recipient gender combination, time from diagnosis to transplant, dose intensity, and donor type, have been observed in previous publications on transplantation in Ph+ ALL [35–40].

This study, being retrospective and registry-based, has several limitations, including the risk of selection bias, missing data, and potentially relevant but unavailable data, such as frontline therapy, hematopoietic cell transplantation-specific comorbidity index, and a relatively long treatment era; thus, treatment options that might have impacted results during this period were not captured. Furthermore, given the long study period (2010–2022), temporal changes in treatment strategies, including the increasing use of immunotherapies for which no data were available, may have affected the study outcomes. Finally, MRD assessment was heterogeneous across participating centers, and data regarding the methodologies employed and the sensitivity of MRD detection were unavailable.

In conclusion, in this registry-based retrospective analysis of Ph+ ALL patients receiving a TKI and subsequent HSCT followed by TKI maintenance, we observed no significant differences in transplantation outcomes between patients with or without ACAs. Notably, achieving MRD-negativity pre-HSCT was significantly more common in patients with ACAs. These data may indicate that the administration of a TKI upfront, followed by HSCT, may overcome the poor prognostic influence of the ACA. Future studies should address the impact of ACAs in Ph+ ALL in the era of bispecific and drug-conjugated monoclonal antibodies and CAR-T cell therapies that have revolutionized the treatment paradigm in ALL.

## Supplementary information

Supplemental material

## Data Availability

AN, JEG, and FC had full access to all the data in the study (available upon data-specific request).
