# Correction: Transplantation outcomes in Philadelphia-positive acute lymphoblastic leukemia with additional cytogenetic abnormalities: A study on behalf of the Acute Leukemia Working Party of the European Society for Blood and Marrow Transplantation

**DOI:** 10.1038/s41409-026-03022-9

**Published:** 2026-08-31

**Authors:** Arnon Nagler, Jordi Esteve, Jacques-Emmanuel Galimard, Depei Wu, Erlie Jiang, Nathalie Dhedin, Anne Sirvent, Jan Vydra, Jakob Passweg, Bruno Lioure, Jaime Sanz, Jurjen Versluis, Yajing Xu, Cristina Castilla-Llorente, Rodrigo Martino Bufarull, Victoria Potter, Nour Ben Abdeljelil, Eolia Brissot, Ali Bazarbachi, Bipin Savani, Sebastian Giebel, Mohamad Mohty, Fabio Ciceri

**Affiliations:** 1 Division of Hematology, Sheba Medical Center, Tel Hashomer, Tel Aviv University, Tel Aviv, Israel; 2 Hematology Department, Institute of Cancer & Blood Disorders (ICAMS), Hospital Clinic, IDIBAPS, University of Barcelona, Barcelona, Spain; 3 EBMT Paris Study Unit, Paris, France; 4 First-Affiliated Hospital of Soochow University, Suzhou, China; 5 Institute of Hematology, Chinese Academy of Medical Sciences, Tianjin, China; 6 Hematology Unit for Adolescents and Adults, Saint Louis Hospital, Paris, France; 7 CHU Lapeyronie, Montpellier, France; 8 Institute of Hematology and Blood Transfusion, Prague, Czechia; 9 University Hospital Basel, Basel, Switzerland; 10 ICANS - Institut de cancerologie Strasbourg Europe, Strasbourg, France; 11 Hematology Department, Hospital Universitari i Politècnic La Fe, Valencia Departament de Medicina Universitat de Valencia, CIBERONC, Instituto Carlos III, Madrid, Spain; 12 Erasmus MC Cancer Institute, Rotterdam, The Netherlands; 13 Central South University - Xiangya Hospital, Changsha, China; 14 Gustave Roussy Cancer Campus, Villejuif, France; 15 Hospital Santa Creu i Sant Pau, Barcelona, Spain; 16 Kings College Hospital, London, United Kingdom; 17 Centre National de Greffe de Moelle, Tunis, Tunisia; 18 Service d’Hématologie clinique et thérapie cellulaire, Hôpital Saint-Antoine, APHP, Paris, France; 19 Hematology-Oncology Division, Department of Internal Medicine, American University of Beirut Medical Center, Beirut, Lebanon; 20 Vanderbilt University Medical Center, Nashville, TN USA; 21 Maria Sklodowska-Curie National Research Institute of Oncology, Gliwice Branch, Gliwice, Poland; 22 Sorbonne University, Department of Haematology, Saint Antoine Hospital, INSERM UMR 938, Paris, France; 23 Ospedale San Raffaele, Haematology and BMT, Milano, Italy

**Keywords:** Leukaemia, Cancer genetics

Correction to: *Bone Marrow Transplantation* https://doi.org/10.1038/s41409-026-02970-6, published online 13 July 2026

In this article the title ‘Transplantation outcomes in Philadelphia-positive acute lymphoblastic leukemia with additional cytogenetic abnormalities’ has been updated to ‘Transplantation outcomes in Philadelphia-positive acute lymphoblastic leukemia with additional cytogenetic abnormalities: A study on behalf of the Acute Leukemia Working Party of the European Society for Blood and Marrow Transplantation’.

The original article has been corrected.

