# Supplementary material for: Transplantation outcomes in Philadelphia-positive acute lymphoblastic leukemia with additional cytogenetic abnormalities: A study on behalf of the Acute Leukemia Working Party of the European Society for Blood and Marrow Transplantation
Source: Bone Marrow Transplant. 2026 Jul 13;61(9):1246–56. doi: 10.1038/s41409-026-02970-6 (PMC13569436; doi:10.1038/s41409-026-02970-6)
Supplement: Supplementary file 1 [file 41409_2026_2970_MOESM1_ESM.docx]

**Supplementary Appendix: Contributing Centers:**

First Affiliated Hospital of Soochow University, Suzhou, China; Institute of Hematology, Chinese Academy of Medical Sciences, Tianjin, China; Saint-Louis Hospital, BMT Unit, Paris, France; CHU Lapeyronie, Montpellier, France; Institute of Hematology and Blood Transfusion, Prague, Czechia; University Hospital | Basel, Basel, Switzerland; ICANS - Institut de cancerologie Strasbourg Europe, Strasbourg, France; University Hospital La Fe, Valencia, Spain; Erasmus MC Cancer Institute, Rotterdam, The Netherlands; Central South University - Xiangya Hospital, Changsha, China; Gustave Roussy Cancer Campus, Villejuif, France; Hospital Santa Creu i Sant Pau, Barcelona, Spain; Kings College Hospital London, London, United Kingdom; Centre National de Greffe de Moelle, Tunis, Tunisia; ICO-Hospital Universitari Germans Trias i Pujol, Badalona, Spain; Olomouc University Hospital, Olomouc, Czech Republic; RM Gorbacheva Research Institute, Pavlov University, St. Petersburg, Russian Federation; Cliniques Universitaires St. Luc, Brussels, Belgium; Azienda Ospedaliera Universitaria Integrata Verona, Verona, Italy; Singapore General Hospital, Singapore, Singapore; Antwerp University Hospital (UZA), Antwerpen, Belgium; University Clinical Centre in Gdansk, Gdansk, Poland; University Hospital Gasthuisberg, Leuven, Belgium; University Med. Center, Ljubljana, Slovenia; Christie Hospital Manchester, Manchester, United Kingdom; RVI Newcastle, Newcastle, United Kingdom; The Second Affiliated Hospital of Xi'an Jiaotong University, Xi'an, China; Klinikum Augsburg, Augsburg, Germany; Klinikum Bremen-Mitte, Bremen, Germany; University of Freiburg, Freiburg, Germany; HUCH Comprehensive Cancer Center, Helsinki, Finland; University Hospital Maastricht, Maastricht, The Netherlands; University La Sapienza, Rome, Italy; Hospital Universitario Virgen del Rocio, Sevilla, Spain; Department of Hematology and Transplantology of Lower Silesian Center of Oncology, Wroclaw, Poland; King Hussein Cancer Centre Adult BMT Program, Amman, Jordan; American University of Beirut Medical Center, Beirut, Lebanon; Birmingham Centre for Cellular Therapy and Transplant (BCCTT), Birmingham, United Kingdom of Great Britain and Northern Ireland (the); Departement d'Oncologie, Service d'Hematologie, Geneva, Switzerland; First Affiliated Hospital of Zhejiang University School of Medicine, Hangzhou, China; University of Muenster, Muenster, Germany; Universita Cattolica S. Cuore, Rome, Italy; Klinik fuer Innere Medzin III, Ulm, Germany; Institut Jules Bordet, Brussels, Belgium; Elisabethinen Hospital, Linz, Austria; Fundacion Jimenez Diaz, Madrid, Spain; Hospital Regional de Malaga, Malaga, Spain; CHU Bordeaux, Hopital Haut-Leveque, Pessac, France; Institut de Cancerologie Lucien Neuwirth, Saint Etienne, France; Hospital Clinico de Valencia, Valencia, Spain; University Hospital Aachen, Aachen, Germany; CHRU, Angers, France; Attikon University General Hospital, Athens, Greece; ZSIS Universitaetsklinikum Knappschaftskrankenhaus Bochum GmbH, Bochum, Germany; USD Trapianti di Midollo, Adulti, Brescia, Italy; University Hospital Brno, Brno, Czech Republic; Department of Bone Marrow Transplantation and Oncohematology, Gliwice, Poland; Klinik fuer Innere Medizin C, Greifswald, Germany; Demiroglu Bilim University Istanbul Florence Nightingale Hospital, Istanbul, Turkey; University Medical Center Schleswig-Holstein, Campus Kiel, Kiel, Germany; Leiden University Hospital, Leiden, The Netherlands; IRCCS San Raffaele Hospital Scientific Institute, Milano, Italy; Nijmegen Medical Centre, Nijmegen, The Netherlands; Unita Operativa di Ematologia, Ravenna, Italy; Armed Forces Bone MarrowTransplant Centre, Rawalpindi, Pakistan; Hospital U. Marques de Valdecilla, Santander, Spain; National Hospital of Haematological Diseases, Sofia, Bulgaria; Robert_Bosch_Krankenhaus, Stuttgart, Germany

**Supplemental Table S1A: Pre-HSCT TKI administration**

| **Variables** | **Modalities** | **N=514** | **noACAs** | **ACAs** |
| --- | --- | --- | --- | --- |
|  |  |  | **n=169** | **n=345** |
| TKI given before HCT | Ima | 219 (42.6) | 62 (36.7) | 157 (45.5) |
|  | Ima+Dasa | 47 (9.1) | 12 (7.1) | 35 (10.1) |
|  | Ima+Dasa+Nilo | 3 (0.6) | 3 (1.8) | 0 (0) |
|  | Ima+Dasa+Nilo+Pona | 2 (0.4) | 1 (0.6) | 1 (0.3) |
|  | Ima+Dasa+Pona | 5 (1) | 1 (0.6) | 4 (1.2) |
|  | Ima+Flumatinib | 3 (0.6) | 0 (0) | 3 (0.9) |
|  | Ima+Nilo | 4 (0.8) | 1 (0.6) | 3 (0.9) |
|  | Ima+Nilo+Pona | 1 (0.2) | 0 (0) | 1 (0.3) |
|  | Ima+Pona | 9 (1.8) | 0 (0) | 9 (2.6) |
|  | Ima+Pona+Flumatinib | 1 (0.2) | 0 (0) | 1 (0.3) |
|  | Dasa | 100 (19.5) | 45 (26.6) | 55 (15.9) |
|  | Dasa+Nilo | 1 (0.2) | 1 (0.6) | 0 (0) |
|  | Dasa+Nilo+Pona | 1 (0.2) | 0 (0) | 1 (0.3) |
|  | Dasa+Pona | 8 (1.6) | 4 (2.4) | 4 (1.2) |
|  | Dasa+Pona+Flumatinib | 1 (0.2) | 0 (0) | 1 (0.3) |
|  | Nilo | 56 (10.9) | 17 (10.1) | 39 (11.3) |
|  | Nilo Dasa | 5 (1) | 3 (1.8) | 2 (0.6) |
|  | Nilo Pona | 6 (1.2) | 1 (0.6) | 5 (1.4) |
|  | Olverembatinib | 1 (0.2) | 0 (0) | 1 (0.3) |
|  | Flumatinib | 19 (3.7) | 9 (5.3) | 10 (2.9) |
|  | Pona | 22 (4.3) | 9 (5.3) | 13 (3.8) |

**Supplemental Table S1B: Post HSCT TKI maintenance**

|  |  | **All patients** | **NoACAs** | **ACAs** |
| --- | --- | --- | --- | --- |
| **N** |  | **Estimation (95% CI)** | **Estimation (95% CI)** | **Estimation (95% CI)** |
| **486** | **Use of maintenance TKI (6 mo.)** | 29.5 (25.5 - 33.6) | 29.1 (22.2 - 36.3) | 30.9 (25.9 - 35.9) |
| **486** | **Use of maintenance TKI (12 mo.) -** | 38.4 (34 - 42.7) | 36.7 (29.2 - 44.2) | 39.2 (33.8 - 44.4) |

Abbreviations: ACAs -additional chromosomal abnormalities; noACAs-no additional chromosomal abnormalities; Ima-imatinib; Dasa-dasatinib; Nilo-nilotinib; Pona-ponatinib; TKI-tyrosine kinase inhibitor; mo.-months; CI- confidence interval; Results expressed as frequency (%)

**Supplemental Table S2: Conditioning regimen details**

| Variables | Modalities | N=514 | No ACAs | ACAs |
| --- | --- | --- | --- | --- |
|  |  |  | n=169 | n=345 |
| Conditioning regimen | TBI-based | 247 (48.1) | 68 (40.2) | 179 (51.9) |
|  | BuCy-based | 127 (24.7) | 58 (34.3) | 69 (20) |
|  | BuFlu-based | 42 (8.2) | 12 (7.1) | 30 (8.7) |
|  | BuFluThio-based | 35 (6.8) | 14 (8.3) | 21 (6.1) |
|  | BuMel-based | 4 (0.8) | 1 (0.6) | 3 (0.9) |
|  | FluMel-based | 40 (7.8) | 12 (7.1) | 28 (8.1) |
|  | TreoFlu | 1 (0.2) | 1 (0.6) | 0 (0) |
|  | Other | 18 (3.5) | 3 (1.8) | 15 (4.3) |

Abbreviations: ACAs -additional chromosomal abnormalities; noACAs-no additional chromosomal abnormalities; TBI-total body irradiation; Mel-melphalan; Bu-busulfan; Flu-fludarabine; Thio-thiotepa; Cy-cytoxan; Treo-treosulfan. Results expressed as frequency (%)

**Supplemental Table S3: GVHD prophylaxis details**

| Variables | Modalities | N=514 | NoACAs | ACAs |
| --- | --- | --- | --- | --- |
|  |  |  | n=169 | n=345 |
| GVHD prophylaxis | CSA | 57 (11.3) | 16 (9.6) | 41 (12.1) |
|  | CSA+MMF | 79 (15.6) | 19 (11.4) | 60 (17.6) |
|  | CSA+MMF+MTX | 137 (27.1) | 57 (34.3) | 80 (23.5) |
|  | CSA+MTX | 165 (32.6) | 56 (33.7) | 109 (32.1) |
|  | CSA+TACRO | 1 (0.2) | 1 (0.6) | 0 (0) |
|  | MMF+MTX+TACRO | 10 (2) | 0 (0) | 10 (2.9) |
|  | MMF+SIRO | 10 (2) | 4 (2.4) | 6 (1.8) |
|  | MMF+TACRO | 21 (4.2) | 7 (4.2) | 14 (4.1) |
|  | MTX-CSA | 1 (0.2) | 0 (0) | 1 (0.3) |
|  | MTX+TACRO | 10 (2) | 2 (1.2) | 8 (2.4) |
|  | PTCy alone | 5 (1) | 1 (0.6) | 4 (1.2) |
|  | SIRO | 1 (0.2) | 0 (0) | 1 (0.3) |
|  | TACRO | 6 (1.2) | 3 (1.8) | 3 (0.9) |
|  | TACRO+SIRO | 3 (0.6) | 0 (0) | 3 (0.9) |
|  | missing | 8 | 3 | 5 |

Abbreviations: ACAs -additional chromosomal abnormalities; noACAs-no additional chromosomal abnormalities; GVHD- graft-versus-host disease; CSA- cyclosporine A; MTX- methotrexate; MMF- mycophenolate mofetil; SIRO- sirolimus; TACRO- tacrolimus; PTCy-post transplant cyclophosphamide. Results expressed as frequency (%)

**Supplemental Table S4: Univariable analysis**

| **Variables** | **Modalities** | **2y OS** | **2y LFS** | **2y RI** | **2y NRM** | **100d AGVH II-IV** | **100d AGVH III-IV** | **2y CGVH** | **2y CGVH ext** | **2y GRFS** |
| --- | --- | --- | --- | --- | --- | --- | --- | --- | --- | --- |
| Nbr of abnormalities | 1_One | 85.2 [78.7-89.8] | 74 [66.5-80.1] | 14.3 [9.4-20.3] | 11.6 [7.3-17.1] | 31 [24.1-38.1] | 9.7 [5.8-14.8] | 39.9 [32.3-47.3] | 20.1 [14.4-26.6] | 53.1 [45.1-60.4] |
|  | 2_Two | 86.2 [78.1-91.4] | 74.9 [65.5-82.1] | 15.9 [9.7-23.5] | 9.2 [4.7-15.6] | 25.5 [17.6-34.1] | 11.3 [6.2-18.2] | 40.7 [31.2-50] | 11.5 [6.2-18.5] | 60.3 [50.3-68.9] |
|  | 3_Complex 3 | 84.8 [76.1-90.6] | 75.8 [66.1-83.1] | 12.2 [6.6-19.5] | 12 [6.6-19.3] | 24.5 [16.5-33.4] | 3.1 [0.8-8] | 39.9 [30.1-49.5] | 19.5 [12.3-27.9] | 55.7 [45.4-64.8] |
|  | 5_Complex 5 | 79.2 [71.3-85.1] | 71.9 [63.5-78.7] | 14.1 [8.8-20.6] | 14 [8.8-20.4] | 28.6 [21.1-36.4] | 10.5 [6-16.4] | 29.6 [22-37.5] | 17 [11.1-24] | 52.2 [43.3-60.4] |
|  | P value | 0.33 | 0.64 | 0.68 | 0.33 | 0.54 | 0.24 | 0.18 | 0.32 | 0.66 |
| Phi isolated | Iso | 85.2 [78.7-89.8] | 74 [66.5-80.1] | 14.3 [9.4-20.3] | 11.6 [7.3-17.1] | 31 [24.1-38.1] | 9.7 [5.8-14.8] | 39.9 [32.3-47.3] | 20.1 [14.4-26.6] | 53.1 [45.1-60.4] |
|  | Not iso | 83 [78.6-86.6] | 74 [69-78.3] | 14.1 [10.7-18.1] | 11.9 [8.8-15.6] | 26.4 [21.8-31.2] | 8.6 [5.9-11.9] | 36.1 [30.9-41.2] | 16 [12.2-20.1] | 55.8 [50.3-60.9] |
|  | P value | 0.49 | 0.98 | 0.92 | 0.87 | 0.29 | 0.48 | 0.63 | 0.53 | 0.8 |
| Complex 3 abn | No | 85.6 [80.9-89.3] | 74.4 [68.7-79.2] | 14.9 [11-19.5] | 10.7 [7.3-14.7] | 28.8 [23.5-34.3] | 10.3 [7.1-14.3] | 40.2 [34.3-46.1] | 16.7 [12.5-21.5] | 55.9 [49.7-61.6] |
|  | Complex3 | 81.6 [76-86] | 73.5 [67.4-78.7] | 13.3 [9.3-18] | 13.2 [9.2-17.8] | 26.8 [21.3-32.7] | 7.4 [4.5-11.2] | 33.9 [27.8-40.1] | 18 [13.3-23.3] | 53.7 [47.1-59.9] |
|  | P value | 0.55 | 0.73 | 0.48 | 0.25 | 0.9 | 0.23 | 0.31 | 0.39 | 0.53 |
| Complex 5 abn | No | 85.4 [81.4-88.6] | 74.8 [70-78.9] | 14.2 [10.8-18] | 11 [8.1-14.4] | 27.7 [23.2-32.3] | 8.4 [5.9-11.5] | 40.1 [35.1-45.2] | 17.5 [13.8-21.5] | 55.9 [50.6-60.8] |
|  | Complex5 | 79.2 [71.3-85.1] | 71.9 [63.5-78.7] | 14.1 [8.8-20.6] | 14 [8.8-20.4] | 28.6 [21.1-36.4] | 10.5 [6-16.4] | 29.6 [22-37.5] | 17 [11.1-24] | 52.2 [43.3-60.4] |
|  | P value | 0.09 | 0.24 | 0.95 | 0.16 | 0.63 | 0.63 | **0.03** | 0.94 | 0.34 |
| Additional derivative t(9;22) | No | 84.4 [80.3-87.7] | 73.6 [68.8-77.8] | 14.9 [11.5-18.7] | 11.5 [8.5-15] | 27.9 [23.4-32.5] | 9.1 [6.5-12.3] | 40.8 [35.7-45.8] | 19.4 [15.5-23.6] | 54.4 [49.2-59.4] |
|  | Yes | 81.9 [74.3-87.5] | 75.1 [66.8-81.6] | 12.2 [7.3-18.4] | 12.7 [7.7-19] | 27.9 [20.4-35.8] | 8.5 [4.5-14.2] | 27.6 [20.2-35.5] | 11.6 [6.8-17.8] | 56.2 [47.2-64.2] |
|  | P value | 0.99 | 0.61 | 0.19 | 0.47 | 1 | 0.91 | **0.03** | 0.28 | 0.99 |
| mono7 or del(7q) | No | 83.9 [80.1-87] | 73.6 [69.1-77.4] | 14.5 [11.3-17.9] | 12 [9.2-15.2] | 29.2 [25-33.5] | 9.4 [6.9-12.4] | 36.2 [31.7-40.7] | 17.7 [14.3-21.5] | 54.3 [49.5-58.9] |
|  | Yes | 83.2 [71.7-90.3] | 77.2 [65-85.6] | 12.2 [5.7-21.5] | 10.6 [4.6-19.4] | 19.7 [11.1-30.1] | 6.1 [1.9-13.6] | 45.4 [32.4-57.5] | 14.7 [7.2-24.8] | 58.8 [45.6-69.8] |
|  | P value | 0.6 | 0.53 | 0.35 | 0.89 | 0.07 | 0.3 | 0.21 | 0.68 | 0.52 |
| IKZF1 or CDKN2A/B | No | 83.8 [80.1-86.9] | 74.3 [70-78] | 13.7 [10.7-17] | 12 [9.2-15.1] | 27.2 [23.2-31.3] | 8.9 [6.5-11.7] | 38.1 [33.6-42.5] | 17.6 [14.3-21.3] | 54.6 [50-59.1] |
|  | Yes | 83.1 [67.6-91.6] | 71 [54.5-82.5] | 19 [8.8-32.2] | 9.9 [3.1-21.7] | 35.7 [21.5-50.2] | 9.5 [3-20.7] | 29.1 [16-43.6] | 14.3 [5.7-26.7] | 58.1 [42.1-71.2] |
|  | P value | 0.98 | 0.88 | 0.48 | 0.56 | 0.363 | 0.99 | 0.14 | 0.44 | 0.39 |
| MRD | Negative | 88.4 [83.8-91.8] | 77.9 [72.3-82.5] | 13.6 [9.8-18.2] | 8.5 [5.5-12.3] | 25.4 [20.2-30.9] | 9 [5.9-12.9] | 36.9 [30.9-42.8] | 15.7 [11.5-20.5] | 59.5 [53.2-65.2] |
|  | Positive | 80.3 [74.5-84.9] | 71.1 [64.7-76.5] | 15 [10.7-20] | 13.9 [9.8-18.7] | 30.8 [24.9-36.8] | 9.2 [5.9-13.4] | 38.2 [31.8-44.5] | 18.8 [14-24.2] | 50.9 [44.2-57.2] |
|  | P value | **0.03** | **0.02** | 0.39 | **0.04** | 0.36 | 0.71 | 0.83 | 0.4 | **0.04** |
| Patient sex | Male | 84.1 [79.2-87.9] | 74.5 [68.9-79.3] | 13.6 [9.8-17.9] | 11.9 [8.4-16] | 26.6 [21.5-31.9] | 9.6 [6.5-13.5] | 40.2 [34.3-46] | 18.4 [14-23.2] | 54 [47.9-59.7] |
|  | Female | 83.4 [77.9-87.6] | 73.4 [67.1-78.6] | 14.9 [10.6-19.9] | 11.7 [8-16.2] | 29.5 [23.7-35.5] | 8.2 [5.1-12.2] | 33.8 [27.7-40.1] | 16.1 [11.6-21.2] | 56 [49.3-62.2] |
|  | P value | 0.99 | 0.86 | 0.98 | 0.79 | 0.35 | 0.57 | 0.21 | 0.57 | 0.57 |
| Female donor to male patient | No | 83.3 [79.4-86.5] | 73.9 [69.5-77.8] | 13.7 [10.6-17.2] | 12.4 [9.5-15.7] | 28.4 [24.2-32.8] | 9.4 [6.9-12.4] | 34.8 [30.2-39.4] | 15.5 [12.3-19.2] | 56.3 [51.4-60.9] |
|  | Yes | 86.2 [76.5-92.1] | 74.7 [63.5-82.9] | 16.6 [9.3-25.7] | 8.8 [3.8-16.2] | 25 [15.9-35.2] | 6.6 [2.4-13.7] | 50.8 [39.1-61.4] | 26.9 [17.5-37.1] | 47.4 [36-58] |
|  | P value | 0.74 | 0.67 | 0.51 | 0.93 | 0.59 | 0.55 | **0.01** | **0.02** | 0.21 |
| Myeloablative | No | 76 [66.6-83.1] | 62.6 [52.5-71.1] | 22.2 [14.7-30.6] | 15.3 [9.2-22.9] | 18.3 [11.5-26.3] | 8.7 [4.2-15.1] | 27.9 [19.6-36.9] | 13.5 [7.7-20.9] | 45.3 [35.6-54.6] |
|  | Yes | 85.7 [81.9-88.8] | 76.9 [72.5-80.8] | 12.1 [9.1-15.6] | 10.9 [8.1-14.2] | 30.4 [26-35] | 9.1 [6.5-12.1] | 39.6 [34.7-44.4] | 18.4 [14.7-22.4] | 57.3 [52.3-62] |
|  | P value | **0.006** | **0.001** | **0.003** | 0.5 | **0.01** | 0.73 | **0.03** | 0.3 | **0.02** |
| TBI | No | 82.8 [77.6-86.9] | 74.6 [68.9-79.5] | 13.2 [9.4-17.7] | 12.1 [8.5-16.4] | 22.8 [18-28.1] | 9.5 [6.4-13.5] | 33.4 [27.7-39.2] | 13.1 [9.3-17.5] | 58.5 [52.2-64.2] |
|  | Yes | 84.8 [79.7-88.8] | 73.3 [67.3-78.4] | 15.2 [11-20] | 11.5 [7.8-15.8] | 33.5 [27.6-39.5] | 8.4 [5.3-12.3] | 41.6 [35.2-47.8] | 22 [17-27.5] | 51 [44.5-57.1] |
|  | P value | 0.58 | 0.48 | 0.62 | 0.65 | **0.003** | 0.84 | 0.06 | **0.002** | 0.051 |
| Cond regimen | MAC-chemo | 84.9 [78.9-89.3] | 79.2 [72.6-84.5] | 9 [5.4-13.8] | 11.8 [7.6-16.8] | 26.3 [20.2-32.7] | 10.2 [6.4-15] | 37.2 [30.3-44.2] | 14 [9.5-19.5] | 62.4 [54.9-69] |
|  | MAC-TBI | 86.5 [81.1-90.4] | 74.9 [68.5-80.2] | 14.9 [10.5-20] | 10.2 [6.6-14.7] | 34.1 [27.8-40.5] | 8.1 [4.9-12.2] | 41.6 [34.8-48.2] | 22.2 [16.8-28.1] | 52.9 [45.9-59.3] |
|  | Non MAC | 76 [66.6-83.1] | 62.6 [52.5-71.1] | 22.2 [14.7-30.6] | 15.3 [9.2-22.9] | 18.3 [11.5-26.3] | 8.7 [4.2-15.1] | 27.9 [19.6-36.9] | 13.5 [7.7-20.9] | 45.3 [35.6-54.6] |
|  | P value | **0.02** | **0.002** | **0.004** | 0.79 | **0.004** | 0.82 | 0.08 | **0.07** | **0.007** |
| Patient CMV | Negative | 86.6 [80.4-91] | 76.2 [68.9-82] | 14.7 [9.7-20.6] | 9.1 [5.3-14.1] | 34.4 [27.1-41.8] | 7.5 [4.1-12.3] | 42.2 [34.5-49.7] | 20.5 [14.6-27.1] | 55.4 [47.4-62.8] |
|  | Positive | 83.2 [78.1-87.2] | 70.9 [65-76] | 16.2 [12-20.9] | 12.9 [9.2-17.3] | 25.1 [20-30.5] | 9.5 [6.3-13.4] | 33.8 [28.1-39.6] | 16 [11.8-20.8] | 53.1 [46.8-58.9] |
|  | P value | 0.11 | 0.39 | 0.82 | 0.19 | **0.047** | 0.55 | 0.09 | 0.3 | 0.82 |
| Age at HSCT (median) | 42 or less | 88.4 [83.8-91.7] | 79 [73.5-83.5] | 11 [7.5-15.2] | 10 [6.7-14] | 32.3 [26.7-38.1] | 9.4 [6.2-13.3] | 38.9 [32.9-45] | 17.7 [13.2-22.6] | 58.7 [52.4-64.5] |
|  | 43 or more | 78.9 [73.3-83.5] | 68.8 [62.6-74.2] | 17.5 [13-22.5] | 13.7 [9.8-18.3] | 23.3 [18.2-28.7] | 8.6 [5.5-12.5] | 35.7 [29.7-41.7] | 17 [12.5-22] | 50.9 [44.5-57] |
|  | P value | **<0.001** | **<0.001** | **0.03** | **0.02** | **0.03** | 0.89 | 0.46 | 0.84 | **0.01** |
| Year of HSCT (median) | 2017 or less | 77 [70.8-82.1] | 64.6 [57.8-70.5] | 19.3 [14.4-24.8] | 16.1 [11.5-21.3] | 31.9 [25.7-38.3] | 11 [7.2-15.6] | 43.7 [36.8-50.3] | 24 [18.4-30] | 41.7 [35.1-48.2] |
|  | 2018 or more | 88.8 [84.6-92] | 81 [75.9-85.1] | 10.4 [7.1-14.3] | 8.6 [5.7-12.2] | 25 [20.2-30.1] | 7.5 [4.9-11] | 32.7 [27.4-38.2] | 12.5 [9-16.6] | 64.7 [58.8-70] |
|  | P value | **<0.001** | **<0.001** | **<0.001** | **0.02** | 0.09 | 0.21 | **0.01** | **0.003** | **<0.001** |
| Source of cells | BM | 86.7 [77.2-92.4] | 83.1 [73.1-89.6] | 8.5 [3.7-15.8] | 8.4 [3.7-15.7] | 30.7 [21-40.9] | 12.3 [6.2-20.4] | 42 [31.1-52.5] | 17.1 [9.8-26] | 62.7 [51.4-72.1] |
|  | PB | 83.2 [79.3-86.4] | 72.2 [67.7-76.3] | 15.3 [12-18.9] | 12.5 [9.5-15.8] | 27.4 [23.2-31.7] | 8.3 [5.9-11.2] | 36.5 [31.8-41.1] | 17.4 [13.9-21.3] | 53.3 [48.3-57.9] |
|  | P value | 0.07 | **0.045** | 0.11 | 0.36 | 0.83 | 0.38 | 0.24 | 0.8 | 0.07 |
| Donor type | Matched rel donor | 86.5 [80.2-90.9] | 69.8 [62.1-76.3] | 21.6 [15.6-28.3] | 8.5 [4.9-13.5] | 22.8 [16.7-29.6] | 6.8 [3.6-11.4] | 41.2 [33.5-48.7] | 23.3 [17-30.1] | 46 [38.1-53.5] |
|  | Mismatched rel donor | 86.6 [80.5-90.9] | 84.2 [77.7-88.9] | 5.4 [2.6-9.6] | 10.4 [6.4-15.6] | 24.6 [18.4-31.3] | 9.4 [5.6-14.3] | 36 [28.8-43.3] | 8.9 [5.2-13.8] | 70.6 [63.1-76.9] |
|  | UD | 78.4 [71.5-83.9] | 67.9 [60.3-74.3] | 15.9 [10.8-21.8] | 16.3 [11.2-22.2] | 36.1 [28.9-43.3] | 10.7 [6.6-15.9] | 35 [27.8-42.3] | 20.3 [14.5-26.7] | 47.7 [40-55] |
|  | P value | **0.03** | **0.001** | **0.0004** | 0.054 | **0.01** | 0.49 | 0.42 | **0.002** | **<0.001** |
| In vivo TCD | No | 87.4 [81.5-91.5] | 74 [66.7-79.9] | 17.4 [12.2-23.5] | 8.6 [5-13.3] | 27.5 [21-34.3] | 8.2 [4.7-12.9] | 45.5 [37.9-52.9] | 28.2 [21.6-35.2] | 44.1 [36.5-51.4] |
|  | Yes | 81.1 [76.3-85] | 73.6 [68.4-78.1] | 12.3 [9-16.2] | 14.1 [10.5-18.1] | 28.3 [23.4-33.3] | 9.4 [6.5-12.9] | 33.2 [28-38.5] | 11.8 [8.5-15.6] | 60.1 [54.5-65.3] |
|  | P value | 0.25 | 0.93 | 0.19 | 0.19 | 0.82 | 0.66 | **0.007** | **<0.001** | **<0.001** |

Abbreviations: HSCT-hematopoietic stem cell transplantation; y-year; RI – relapse incidence; NRM – non-relapse mortality; LFS – leukemia-free survival; OS - overall survival; AGVH – acute graft-versus-host disease; CGVH – chronic graft-versus-host disease; ext-extensive; GRFS - GVHD-free, relapse-free survival; mono-monosomy; Nbr-number; Phi-Philadelphia; iso-isolated; abn- abnormality; del-deletion; CMV- cytomegalovirus; rel-related; UD-unrelated donor; TBI-total body irradiation; MRD-measurable residual disease; cond-conditioning; BM-bone marrow; PB-peripheral blood; MAC-myeloablative; TCD- T-cell depletion
